# Palmitate- and C6 ceramide-induced *Tnnt3* pre-mRNA alternative splicing occurs in a PP2A dependent manner

**DOI:** 10.1186/s12986-018-0326-3

**Published:** 2018-12-17

**Authors:** Adam J. Black, Rudolf J. Schilder, Scot R. Kimball

**Affiliations:** 1 Department of Cellular and Molecular Physiology, Penn State College of Medicine, H166, 500 University Drive, Hershey, PA 17033 USA; 2 Present Address: Department of Cell Biology and Physiology, 6330 Medical Biomolecular Research Building, 111 Mason Farm Rd, Chapel Hill, NC 27599 USA; 3 Department of Entomology and Biology, Penn State University, University Park, PA USA

**Keywords:** Alternative splicing, Fatty acids, Palmitate, Ceramides, Troponin T, *Tnnt3*, Protein phosphatase 2A

## Abstract

**Background:**

In a previous study, we showed that consumption of diets enriched in saturated fatty acids causes changes in alternative splicing of pre-mRNAs encoding a number of proteins in rat skeletal muscle, including the one encoding skeletal muscle Troponin T (*Tnnt3*). However, whether saturated fatty acids act directly on muscle cells to modulate alternative pre-mRNA splicing was not assessed. Moreover, the signaling pathway through which saturated fatty acids act to promote changes in alternative splicing is unknown. Therefore, the objective of the present study was to characterize the signaling pathway through which saturated fatty acids act to modulate *Tnnt3* alternative splicing.

**Methods:**

The effects of treatment of L6 myotubes with saturated (palmitate), mono- (oleate), or polyunsaturated (linoleate) fatty acids on alternative splicing of pre-mRNA was assessed using *Tnnt3* as a marker gene.

**Results:**

Palmitate treatment caused a two-fold change (*p* < 0.05) in L6 myotube *Tnnt3* alternative splicing whereas treatment with either oleate or linoleate had minimal effects compared to control myotubes. Treatment with a downstream metabolite of palmitate, ceramide, had effects similar to palmitate on *Tnnt3* alternative splicing and inhibition of de novo ceramide biosynthesis blocked the palmitate-induced alternative splicing changes. The effects of palmitate and ceramide on *Tnnt3* alternative splicing were accompanied by a 40–50% reduction in phosphorylation of Akt on S473. However, inhibition of de novo ceramide biosynthesis did not prevent palmitate-induced Akt dephosphorylation, suggesting that palmitate may act in an Akt-independent manner to modulate *Tnnt3* alternative splicing. Instead, pre-treatment with okadaic acid at concentrations that selectively inhibit protein phosphatase 2A (PP2A) blocked both palmitate- and ceramide-induced changes in *Tnnt3* alternative splicing, suggesting that palmitate and ceramide act through PP2A to modulate *Tnnt3* alternative splicing.

**Conclusions:**

Overall, the data show that fatty acid saturation level and ceramides are important factors modulating alternative pre-mRNA splicing through activation of PP2A.

**Electronic supplementary material:**

The online version of this article (10.1186/s12986-018-0326-3) contains supplementary material, which is available to authorized users.

## Introduction

Alternative splicing is a critical step in the processing of precursor mRNA (pre-mRNA) into mature mRNA that involves the selective removal of exons from a pre-mRNA. Alternative splicing can generate multiple mature mRNAs from a single gene thus allowing for a significant expansion of the transcriptome and a concomitant increase in proteome plasticity and functionality [1]. The regulation of alternative splicing is complex. In brief, it involves the dynamic interplay of *cis*- and *trans*-acting factors to coordinate the placement of the spliceosome, a large macromolecular complex responsible for the catalytic removal of introns and exons, onto a pre-mRNA [2–6]. *Cis*-acting factors are RNA sequence elements and thus are inherent to the pre-mRNA [7, 8], whereas *trans*-acting factors are splicing accessory proteins that bind *cis*-factors to facilitate splice site recognition [6, 9, 10]. In addition to being regulated through transcriptional mechanisms, *trans*-acting factors are also post-translationally modified by kinases and phosphatases [11, 12]. Thus, the phosphorylation state of components of the splicing regulatory machinery is a critical determinant of whether or not an alternatively spliced exon is included in or excluded from a mature RNA.

One of the best characterized examples of phosphorylation-mediated regulation of alternative splicing is through the Akt signaling axis. Indeed, several *trans-*acting factors (i.e. serine-arginine-rich (SR) proteins and SR protein kinases) involved in pre-mRNA alternative splicing are substrates of Akt [4, 11–14]. The contribution of protein phosphatases (PP) to changes in *trans*-acting factor phosphorylation is less well studied. Protein phosphatases (PP)1 and 2A are required for the proper assembly and disassembly of spliceosomal components [15] and regulate alternative splicing by directly targeting *trans*-acting proteins or indirectly by modulating Akt phosphorylation [16, 17].

Troponin is a heterotrimeric complex that mediates calcium-induced sarcomere contraction [18]. The troponin T subunit links the calcium binding subunit, troponin C, to tropomyosin, and therefore plays a critical role in calcium signaling to the sarcomere. We recently demonstrated that fast skeletal muscle troponin T (*Tnnt3*) pre-mRNA is alternatively spliced in rat gastrocnemius muscle in response to consumption of a high-fat diet prior to the onset of obesity. Interestingly, this effect was specific to diets enriched in lard, as diets enriched in mono- or polyunsaturated fatty acids had no effect on *Tnnt3* alternative splicing [19]. A major difference between the three diets used in that study was the proportion of the saturated fat, palmitate, in the lard-enriched diet. Palmitate is a substrate commonly used in the biosynthesis of ceramides [20, 21], a family of sphingolipid second messengers that are induced in response to a variety of cellular stressors [22–24]. The above observations suggest that the diet-specific effects on *Tnnt3* pre-mRNA alternative splicing are caused by specific types of fatty acids in the high-fat lard diet and/or the intramuscular accumulation of ceramides. There is some precedent for this hypothesis, as previous work demonstrated that PP1 is necessary for the ceramide-induced changes in the alternative splicing of Bcl-xL and caspase-9 pre-mRNAs [24]. Other studies have reported diet- or obesity-induced changes in pre-mRNA alternative splicing [25–34], but little is known about the role of specific fatty acids in this process.

Though it is tempting to speculate that changes in skeletal muscle alternative splicing in the aforementioned studies were due to the accumulation of fatty acids or ceramides, hormonal feedback mechanisms controlling metabolic status complicate delineating mechanisms that modulate alternative splicing in vivo [1, 35]. Hence, we employed an in vitro system to precisely control the exposure of muscle cells to fatty acids and ceramides to examine the effects on alternative splicing and potential signaling pathways involved in regulating this process. Therefore, using *Tnnt3* as a marker gene in L6 myotubes we hypothesized that treatment with saturated fatty acids and ceramides would alter the pattern of alternative splicing while unsaturated fatty acids would have little to no effect.

## Methods

### Fatty acid/BSA conjugation

Palmitate, oleate, or linoleate (Sigma, St. Louis, MO) were dissolved in glass vials at 70 °C in a 50% Ethanol/Dulbecco’s phosphate buffered saline (DPBS) (Gibco, Thermo Fisher, Waltham, MA) solution and added to a solution of 10% fatty acid-free BSA (Sigma) that was pre-warmed to 55 °C. The fatty acid-BSA conjugates were mixed at 55 °C for 20 min then filter sterilized through 0.45 μm syringe filters (Millipore, Billerica, MA) and stored at − 20 °C in glass vials.

### Cell culture

L6 myoblasts (1 × 10^5^; passage 3–10) (ATCC, Manassas, VA) were seeded in six-well culture plates in DMEM (Gibco) containing 10% FBS (Atlas Biologicals, Fort Collins, CO) and 1% Pen/Strep (Gibco). When the L6 myoblasts reached 80–85% confluence (approximately 48 h post seeding) they were induced to differentiate to myotubes by replacing the media with DMEM containing 2% horse serum (Sigma) and 1% Pen/Strep (differentiation media (DM)). DM was refreshed every 48 h. At the end of the sixth day, fresh DM was added approximately 16 h prior to the addition of fatty acids, C6 ceramide (d18:1/6:0) (Avanti Polar Lipids, Alabaster, Alabama), or chemical inhibitors of de novo ceramide biosynthesis (myriocin) or PP2A (okadaic acid). For experiments using chemical inhibitors, L6 myotubes were pretreated for two hours with 50 nM myriocin (Sigma), 15 nM okadaic acid (LC Labs, Woburn, MA), or vehicle (methanol or DMSO, respectively) prior to the addition of 150 μM fatty acid-BSA conjugate or 20 μM C6 ceramide. Cells were treated with fatty acid-BSA conjugate or C6 ceramide for 24 h and collected for analysis eight days after the onset of differentiation.

### Western blot analysis

L6 myotubes were washed once in cold phosphate buffered saline and harvested in lysis buffer as described previously [36]. Myotube samples were fractionated by SDS-PAGE using Bio-Rad precast Criterion gels (Bio-Rad, Hercules, CA) as previously described [37]. Select PVDF membranes were stained with a reversible protein stain kit according to the manufacturer’s instructions (Pierce, Thermo Fisher). Primary antibodies against phospho-Akt (S473) (#4060; 1:2000), ERK1/2 (T202/Y204) (#9101; 1:1000), and GSK3β (S9) (#5558; 1:1000), and total-Akt (#9272; 1:1000), ERK1/2 (#9102; 1:1000), and GSK3β (#12456; 1:1000) were purchased from Cell Signaling (Danvers, MA). Anti-TNNT3 antibody (#JLT12; 0.2 μg/mL) was purchased from Developmental Studies Hybridoma Bank (Iowa City, IA). Western blot densitometry was quantitated using Image J Software (National Institutes of Health). Densitometry was normalized to a pooled sample of untreated myotubes collected in lysis buffer as above and run in duplicate on each gel.

### *Tnnt3* splice form characterization and quantification

L6 myotubes were collected in 1 mL TRIzol then stored at − 80 °C until total RNA was extracted per manufacturer’s instructions (Invitrogen, Thermo Fisher), with the exception that the precipitated RNA pellet underwent an additional 85% ethanol wash. One μg of total RNA was reverse transcribed to cDNA using the High Capacity cDNA Reverse Transcription Kit (Applied Biosystems, Thermo Fisher). PCR was used to amplify *Tnnt3* amplicons representing all expressed splice forms as described previously [36]. Fluorescein (6FAM)-labeled *Tnnt3* PCR products were diluted 1:10 and one μL per sample was analyzed by capillary electrophoresis as described previously [36]. Briefly, separated *Tnnt3* amplicons were detected as individual fluorescence peaks of varying height within a range of 0-1200 bp. An internal size standard allowed accurate sizing of *Tnnt3* amplicons that were determined to vary between ~ 700-800 bp. The relative abundance of each *Tnnt3* amplicon was then calculated by dividing the fluorescence peak height by the total of all *Tnnt3* amplicon peak heights. The fold change in the relative abundance of each *Tnnt3* splice form relative to controls was calculated for each experiment.

### Quantitative real-time PCR of *Tnnt3* mRNA

Quantitative real-time PCR (qRT-PCR) was performed using the QuantStudio 12 K Flex Real-Time PCR System and SYBR Green master mix (Qiagen, Germantown, MD) according to the manufacturer’s instructions. *Tnnt3* gene expression was quantified using rat specific primers designed to constitutively expressed (i.e., not alternatively spliced) *Tnnt3* exons 11 and 12 (Fwd: 5’ GTCAGAACAAGGACCTCATGG 3′, Rev.: 5’ TCTCAGCGCGAATCCTTTG 3′) and a region within the gene *Gapdh* (Fwd: 5’-AGTTCAACGGCACAGTCAAG-3′, Rev.: 5’-TACTCAGCACCAGCATCACC-3′). Changes in *Tnnt3* gene expression were normalized to *Gapdh* using the ${2}^{-\Delta  \Delta  {C}_T}$ method as described previously [38].

### Statistical analysis

*Tnnt3* splice form and Western blot analyses are displayed as the mean fold change ± SEM of the relative abundance or ratio of phosphorylated/total protein, respectively, compared to controls. Fold change values were calculated from each of three independent experiments with three replicates per experiment. GraphPad Prism v7.0 was used to generate graphs and to perform Student’s t-tests or One- or Two-way ANOVA with Fisher’s LSD post-hoc test for multiple comparisons to determine statistical significance (*p* ≤ 0.05).

## Results

### Characterization of L6 myotube *Tnnt3* splice form expression

**Fig. 1:**
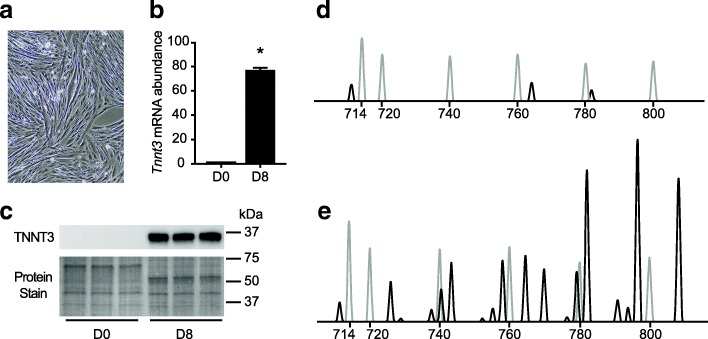
Characterization of *Tnnt3* mRNA, protein, and splice form expression in differentiating L6 myotubes. L6 myotubes were collected at the onset of differentiation (D0) or eight days post differentiation (D8) as described in Methods. (**a**) Micrograph of D8 L6 myotubes. (**b**) *Tnnt3* mRNA abundance was assessed by qRT-PCR and normalized to *Gapdh*. (**c**) TNNT3 protein expression was assessed by Western blot analysis. Protein staining of the Western blot membrane is shown to demonstrate equal protein loading. Capillary electrophoresis tracings of *Tnnt3* splice forms from (**d**) D0 or (**e**) D8 of differentiation as assessed by fragment analysis of *Tnnt3* PCR amplicons. Black and gray tracings represent *Tnnt3* splice forms and internal size standards, respectively. Peak height is proportional to the splice form relative abundance. Data in (**b**) are presented as means ± SEM from three independent experiments using three replicates per time point. Statistically different means as assessed by Student’s t-test are denoted with an asterisk (*) (*p* ≤ 0.05)

Skeletal muscle contractile elements, including *Tnnt3*, are expressed in differentiated mouse (C2C12) myotubes [36, 39], but to our knowledge a comprehensive analysis of *Tnnt3* splice forms had not been performed previously in rat L6 myotubes. Thus, we induced L6 myoblasts to differentiate into myotubes and analyzed *Tnnt3* total mRNA, protein, and splice form expression at day 0 (D0), and day 8 (D8) post-differentiation onset. Myoblasts fused to form mature myotubes (Fig. 1a) and total *Tnnt3* mRNA abundance, as assessed by qRT-PCR, was increased 76-fold by D8 (Fig. 1b). A similar effect was observed with TNNT3 protein expression (Fig. 1c). Capillary electrophoresis of *Tnnt3* PCR amplicons detected three *Tnnt3* splice forms present in low abundance at D0 (Fig. 1d). Expression of all three splice forms present in undifferentiated myoblasts increased after differentiation, and 15 splice forms not detected in myoblasts were present in D8 myotubes (Fig. 1e).

### In vitro modulation of *Tnnt3* alternative splicing by fatty acids and ceramides

**Fig. 2:**
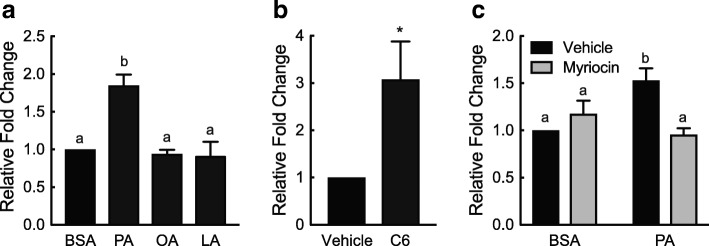
Fatty acid- and C6 ceramide-induced changes in *Tnnt3* pre-mRNA alternative splicing. L6 myotubes were treated for 24 h with (**a**) 150 μM palmitate, oleate, or linoleate (PA, OA, LA, respectively) conjugated to BSA or an equal volume of BSA alone, (**b**) 20 μM C6 ceramide (C6) or an equal volume of methanol (Vehicle), or (**c**) 50 nM myriocin or an equal volume of methanol (Vehicle) for two hours prior to a 24-h treatment with 150 μM PA bound to BSA (PA) or an equal volume of BSA alone. The fold change in the relative abundance of the 737 base pair *Tnnt3* splice form was assessed by capillary electrophoresis. Data are presented as means ± SEM from three independent experiments using three replicates per treatment. Statistical significance was assessed by Student’s t-test (**b**) and One- and Two-way ANOVA with Fishers LSD post-hoc test for multiple comparisons (**a** and **c**, respectively). Statistically different means are denoted with an asterisk (*) or different letters above the bars (*p* ≤ 0.05)

We next tested if exposure to fatty acids could modulate alternative splicing of *Tnnt3* in L6 myotubes. Cells were treated with BSA conjugated to saturated (palmitate), mono- (oleate) and poly-unsaturated (linoleate) fatty acids that were also present in the diets of our previous study [19], or with BSA alone for 24 h. Treatment with oleate or linoleate significantly altered the expression of the 781 and 795 bp *Tnnt3* splice forms compared to BSA-treated myotubes, however the magnitude of the effect was small (i.e., 1.2-fold or less; Table 1). The splice form exhibiting the largest change was the 737 bp *Tnnt3* splice form in response to palmitate treatment. This effect was specific to palmitate as it caused a 1.8-fold increase in relative abundance compared to treatment with BSA alone while neither oleic nor linoleic acid treatment had any effect on its relative abundance (Fig. 2a and Table 1). The 737 bp *Tnnt3* splice form was the most consistently and robustly changed splice form across studies. Moreover, the most abundant splice form in adult rat loading bearing muscle, i.e. the gastrocnemius, is the 737 bp splice form [34]. Therefore, for ease of presentation, from here onward data for all 18 *Tnnt3* splice forms will be presented in table format as Additional Files and data for the 737 bp *Tnnt3* splice form will be presented in graphical format.

**Table 1:** Fold change in the relative abundance of *Tnnt3* splice forms

*Tnnt3* splice form size (bp)	BSA	PA	OA	LA
710	1.00 ^a^	1.228 ± 0.240 ^a^	1.042 ± 0.099 ^a^	1.091 ± 0.067 ^a^
725	1.00 ^a, c^	1.212 ± 0.109 ^a^	0.970 ± 0.041 ^b, c^	1.022 ± 0.078 ^a, c^
728	1.00 ^a^	0.713 ± 0.098 ^a^	0.832 ± 0.239 ^a^	0.875 ± 0.270 ^a^
737	1.00 ^a^	1.851 ± 0.143 ^b^	0.943 ± 0.053 ^a^	0.921 ± 0.180 ^a^
739	1.00 ^a^	1.438 ± 0.202 ^a^	1.327 ± 0.350 ^a^	1.115 ± 0.403 ^a^
742	1.00 ^a^	1.026 ± 0.057 ^a^	0.946 ± 0.044 ^a^	0.978 ± 0.117 ^a^
751	1.00 ^a^	1.198 ± 0.209 ^a^	0.662 ± 0.247 ^a^	0.794 ± 0.063 ^a^
754	1.00 ^a, b^	1.233 ± 0.177 ^a^	0.764 ± 0.150 ^b^	0.826 ± 0.069 ^b^
757	1.00 ^a^	1.105 ± 0.061 ^a^	0.923 ± 0.091 ^a^	1.013 ± 0.086 ^a^
763	1.00 ^a^	0.989 ± 0.104 ^a^	0.860 ± 0.097 ^a^	0.884 ± 0.069 ^a^
769	1.00 ^a^	1.071 ± 0.036 ^a^	0.906 ± 0.124 ^a^	0.804 ± 0.112 ^a^
775	1.00 ^a^	1.334 ± 0.177 ^a^	0.990 ± 0.233 ^a^	0.977 ± 0.254 ^a^
778	1.00 ^a^	0.999 ± 0.164 ^a^	1.151 ± 0.017 ^a^	1.085 ± 0.119 ^a^
781	1.00 ^a^	0.950 ± 0.030 ^a^	1.104 ± 0.014 ^b^	1.114 ± 0.033 ^b^
790	1.00 ^a^	0.839 ± 0.070 ^a^	0.833 ± 0.042 ^a^	0.790 ± 0.157 ^a^
793	1.00 ^a^	0.837 ± 0.036 ^b, c^	0.935 ± 0.054 ^a, c^	0.776 ± 0.078 ^b^
795	1.00 ^a, b^	0.953 ± 0.044 ^a^	1.071 ± 0.043 ^b^	1.216 ± 0.078 ^c^
807	1.00 ^a^	0.966 ± 0.030 ^a, b^	0.955 ± 0.035 ^a, b^	0.909 ± 0.020 ^b^

L6 myotubes were treated for 24 h with 150 μM palmitate, oleate, or linoleate (PA, OA, LA, respectively) conjugated to BSA or an equal volume of BSA alone. The fold change in the relative abundance of *Tnnt3* splice forms was assessed by capillary electrophoresis. Data are presented as means ± SEM from three independent experiments using three replicates per treatment. Statistical significance was assessed by One-way ANOVA and Fishers LSD post-hoc test for multiple comparisons. Statistically different means are denoted with different letters (*p* ≤ 0.05)

Palmitate is a substrate for serine palmitoyltransferase, the initial and rate limiting step in de novo ceramide biosynthesis [40]. Thus, the palmitate-induced effects on *Tnnt3* alternative splicing may be due to de novo biosynthesis of ceramides. Indeed, a 24-h treatment with 20 μM C6 ceramide increased the relative abundance of the 737 bp *Tnnt3* splice form by 2.7-fold (Fig. 2b and Additional file 1). Additionally, C6 ceramide treatment significantly altered the relative abundance of 6 of the 18 *Tnnt3* splice forms (Additional file 1). Pre-treatment with myriocin, a selective serine palmitoyltransferase inhibitor which blocks the initial, crucial catalytic step of ceramide biosynthesis, attenuated the palmitate-induced increase in the relative abundance of the 737 bp *Tnnt3* splice form (Fig. 2c and Additional file 2). These data demonstrate that both fatty acids and ceramides induce changes in *Tnnt3* alternative splicing and that the effect of palmitate likely requires de novo ceramide synthesis.

### In vitro modulation of Akt S473 phosphorylation by fatty acids and ceramides

**Fig. 3:**
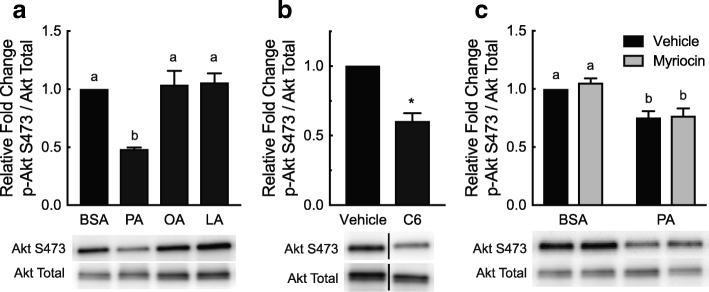
Fatty acid- and C6 ceramide-induced changes in Akt S473 phosphorylation. L6 myotubes were treated for 24 h with (**a**) 150 μM palmitate, oleate, or linoleate (PA, OA, LA, respectively) conjugated to BSA or an equal volume of BSA alone, (**b**) 20 μM C6 ceramide (C6) or an equal volume of methanol (Vehicle), or (**c**) 50 nM myriocin or an equal volume of methanol (Vehicle) for two hours prior to a 24-h treatment with 150 μM PA bound to BSA or an equal volume of BSA alone. The ratio of phosphorylated Akt at S473 to total Akt was assessed by Western blot analysis. Black lines in the Western blot images shown in B represent noncontiguous lanes from the same blot. Data are presented as means ± SEM from three independent experiments using three replicates per treatment. Statistical significance was assessed by Student’s t-test (**b**) and One- and Two-way ANOVA with Fishers LSD post-hoc test for multiple comparisons (**a** and **c**, respectively). Statistically different means are denoted with an asterisk (*) or different letters above the bars (*p* ≤ 0.05)

We next sought to gain insight into possible signaling pathways involved in the palmitate- and ceramide-induced effects on *Tnnt3* alternative splicing. Therefore, we assessed whether Akt signaling correlated with changes to the relative abundance of the 737 bp *Tnnt3* splice form because a hallmark effect of ceramides is their ability to alter Akt phosphorylation [23, 41] and our previous findings in C2C12 myotubes suggested an important role for Akt in modulating *Tnnt3* pre-mRNA alternative splicing [36]. As shown in Fig. 3a, palmitate, but not oleate or linoleate, reduced Akt S473 phosphorylation compared to BSA-treated L6 myotubes (Fig. 3a). Moreover, treatment with C6 ceramide reduced Akt S473 phosphorylation by 39.5 ± 5.6%, compared to controls (Fig. 3b). Interestingly, although palmitate treatment significantly reduced Akt S473 phosphorylation, pretreatment with myriocin did not prevent the palmitate-induced reduction (Fig. 3c), suggesting that palmitate is either not acting through Akt or acts downstream of Akt to modulate alternative splicing.

### Inhibition of PP2A attenuates the palmitate-and ceramide-induced alternative splicing of *Tnnt3*

**Fig. 4:**
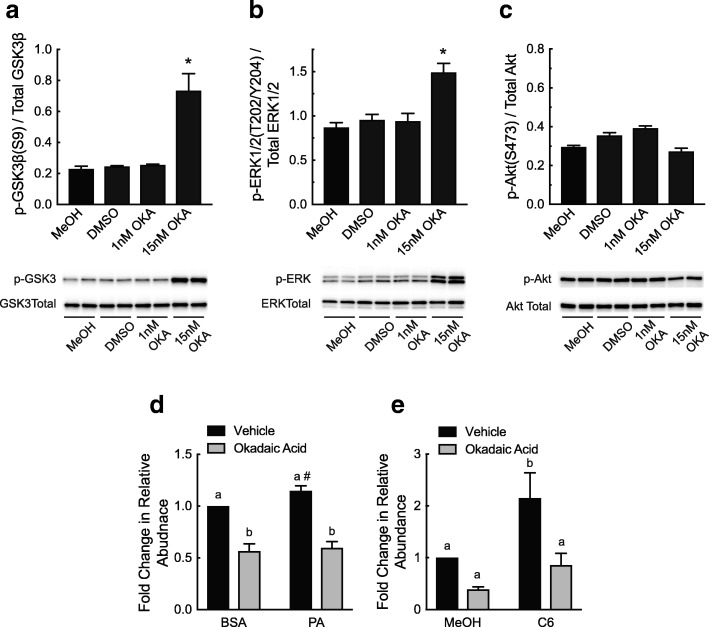
Palmitate- and C6 ceramide-induced changes in *Tnnt3* pre-mRNA alternative splicing are blocked by inhibition of PP2A. **a-c** L6 myotubes were pretreated for two hours with either 1 or 15 nM okadaic acid (OKA) or an equal volume of MeOH or DMSO for 24-h prior to Western blot analysis. The ratio of (**a**) GSK3β at S9 to total GSK3β, (**b**) ERK1/2 at T202/Y204 to total ERK1/2, and (**c**) phosphorylated Akt at S473 to total Akt was assessed. L6 myotubes were pretreated for two hours with okadaic acid or an equal volume of DMSO (Vehicle) prior to a 24-h treatment with (**d**) 150 μM PA conjugated to BSA (PA) or an equal volume of BSA alone or (**e**) 20 μM C6 ceramide (C6) or an equal volume of methanol (MeOH). The fold change in the relative abundance of the 737 base pair *Tnnt3* splice form was assessed by capillary electrophoresis (**d** and **e**). Data are presented as means ± SEM from three independent experiments using three replicates per treatment. Statistical significance was assessed by Student’s t-test (**a**-**c**) or Two-way ANOVA with Fishers LSD post-hoc test for multiple comparisons (**d** and **e**). Statistically different means are denoted with an asterisk (*) or different letters above the bars (*p* ≤ 0.05). # *p* = 0.08 vs. BSA/Vehicle

A major mode of action of ceramides is the activation of PP1 and PP2A [16, 41]. Because changes in Akt S473 phosphorylation, a PP1 specific site [42], did not explain the palmitate-induced effects on *Tnnt3* alternative splicing, we hypothesized that palmitate and C6 ceramide exert their effects on *Tnnt3* alternative splicing in part through the activation of PP2A. A previous study [43] showed that 15 nM okadaic acid selectively inhibited PP2A in primary cultures of rat hepatocytes. Confirming that finding, we found that in L6 myotubes, 15 nM okadaic acid significantly increased the phosphorylation of the PP2A substrates GSK-3β [44] and ERK1/2 [45] but not the PP1 substrate Akt S473 (Fig. 4a-c). Notably, pretreatment with 15 nM okadaic acid blocked the effect of palmitate and C6 ceramide on the abundance of the 737 bp *Tnnt3* splice form (Fig. 4d and e) and prevented or attenuated the change in abundance of several other *Tnnt3* splice forms (Additional files 3 and 4). Thus, these data suggest that the effects of palmitate and C6 ceramide on the alternative splicing of the 737 bp *Tnnt3* splice form expression occurs in a PP2A-dependent manner.

## Discussion

The results of the present study support a model in which saturated fatty acids increase ceramide expression leading to the activation of PP2A and subsequent changes in the alternative splicing of *Tnnt3* pre-mRNA in L6 myotubes. In support of this model, the data show that both palmitate and C6 ceramide promote accumulation of the 737 bp *Tnnt3* splice form and that the effect of palmitate is blocked by the ceramide synthesis inhibitor, myriocin. Although we did not measure ceramide levels in this study, others have demonstrated a time- and dose-dependent increase in ceramide accumulation in palmitate-treated cells in culture [46] and that the increase is blocked by myriocin treatment [20]. Thus, the repressive effect of myriocin on palmitate-induced *Tnnt3* alternative splicing is consistent with the fatty acid acting through a mechanism requiring increased ceramide synthesis to produce changes in alternative splicing. Importantly, previous studies have demonstrated that increased ceramide production is associated with changes in alternative splicing of pre-mRNAs other than *Tnnt3*. For example, both TNFα and gemcitabine have been reported to alter the pattern of pre-mRNA alternative splicing of PKCβII, caspase 9, and Bcl-xL and myriocin blocks the effect [23, 24], suggesting that, like palmitate, TNFα and gemcitabine-induced changes in alternative splicing require de novo ceramide synthesis.

In the present study, we also found that fatty acids of varying saturation level elicit distinct patterns of *Tnnt3* pre-mRNA alternative splicing. Notably, in several instances, the pattern of *Tnnt3* alternative splicing was different in myotubes treated with the unsaturated fatty acids oleate and linoleate, compared to those treated with palmitate. For example, treatment with either oleate or linoleate elicited differential effects on the expression of the 793 and 795 bp *Tnnt3* splice forms (Table 1). The effect of oleate or linoleate is most likely independent of increased ceramide production because, unlike palmitate, they have no effect on ceramide production or accumulation in skeletal muscle [47] or in cells in culture [48–50]. Instead, it is possible that there are ceramide-independent mechanisms that lead to variations in the *Tnnt3* splice form expression in response to treatment with unsaturated fatty acids. However, the magnitude of the changes in alternative splicing induced by unsaturated fatty acids was small, and the physiological significance of such relatively small changes must be questioned.

An important mechanism for regulating alternative splicing involves changes in phosphorylation of the SR proteins that belong to the non-small nuclear ribonucleoprotein particle splicing factor family of proteins [4, 11–14]. The SR proteins are phosphorylated on multiple serine residues by various kinases including Akt, leading to changes in the alternative splicing process [12, 14]. In the present study, Akt phosphorylation on S473 was reduced in response to treatment with either palmitate or C6 ceramide in association with increased *Tnnt3* 737 abundance, consistent with a possible role for altered Akt signaling in mediating the observed changes in alternative splicing. However, although myriocin blocked the palmitate-induced changes in alternative splicing, it did not prevent the changes in Akt S473 phosphorylation, suggesting that the changes in alternative splicing occurred in an Akt-independent manner. Instead, okadaic acid when used at a concentration that inhibits PP2A but not PP1 blocked the effect of both palmitate and ceramide on alternative splicing of the *Tnnt3* 737 splice form. Previous studies have shown that ceramide treatment leads to activation of both PP1 and PP2A [51–53]. Thus, it is tempting to speculate that palmitate-induced ceramide production leads to activation of PP2A and subsequent modulation of *Tnnt3* alternative splicing.

It is interesting that treatment with C6 ceramide had a more dramatic effect on *Tnnt3* alternative splicing compared to palmitate, both with regard to the number of splice forms affected and, in some cases, the magnitude of the change. Although the basis for this difference is unknown, it is noteworthy that C6 ceramide is cell permeable and does not need to be bound to BSA to be soluble in aqueous solution [24]. In contrast, palmitate was bound to BSA and requires fatty acid transport proteins to enter the cell [54]. Thus, C6 ceramide likely enters the cell more rapidly compared to palmitate, and may reach higher intracellular concentrations more quickly compared to the ceramide generated by palmitate-induced increases in synthesis. It is also possible that the shorter chain ceramides like C6 ceramide have a differential effect to promote alternative splicing compared to longer chain ones.

## Conclusion

In addition to acting as energy sources and structural components of cells, fatty acids and their metabolites act as signaling molecules to regulate gene expression. The data presented in the current study extend the biological significance of fatty acids by demonstrating that fatty acids and their metabolites play a role in the selection of exons for alternative splicing and uncover an important node in the nutrient control of gene expression. The basic mechanisms of pre-mRNA alternative splicing have been studied extensively [10, 55]. However, surprisingly, how alternative splicing regulatory mechanisms are influenced by changes in environmental conditions or nutrient availability has only recently been investigated [19, 56, 57]. Approximately 95% of all multi-exon pre-mRNAs are thought to generate multiple mature mRNA splice forms [3], therefore future studies should expand upon the list of pre-mRNAs affected by nutrient availability, the signaling pathways involved, and the functional outcomes of these changes.

## Additional files

Additional file 1: Fold change in the relative abundance of *Tnnt3* splice forms in L6 myotubes treated with C6 ceramide. (DOCX 15 kb)

Additional file 2: Fold change in the relative abundance of *Tnnt3* splice forms in L6 myotubes treated with myriocin. (DOCX 16 kb)

Additional file 3: Fold change in the relative abundance of *Tnnt3* splice forms in L6 myotubes treated with okadaic acid and palmitate. (DOCX 16 kb)

Additional file 4: Fold change in the relative abundance of *Tnnt3* splice forms in L6 myotubes treated with okadaic acid and C6 ceramide. (DOCX 16 kb)

## Abbreviations

Bcl-xL: B-cell lymphoma extra-long
bp: base pairs
BSA: bovine serum albumin
DM: differentiation medium
ERK: extracellular signal-regulated kinase
GSK3β: glycogen synthase kinase 3β
LA: linoleic acid
OA: oleic acid
PA: palmitic acid
PP: protein phosphatase
SR: serine-arginine-rich
TNFα: tumor necrosis factor α
Tnnt3: troponin T3

## References

[1] Baralle FE, Giudice J (2017). Alternative splicing as a regulator of development and tissue identity. Nat Rev Mol Cell Biol..

[2] Bortfeldt R, Schindler S, Szafranski K, Schuster S, Holste D (2008). Comparative analysis of sequence features involved in the recognition of tandem splice sites. BMC Genomics.

[3] Lee Y, Rio DC (2015). Mechanisms and regulation of alternative pre-mRNA splicing. Annu Rev Biochem.

[4] Naro C, Sette C (2013). Phosphorylation-mediated regulation of alternative splicing in cancer. Int J Cell Biol.

[5] Castle JC, Zhang C, Shah JK, Kulkarni AV, Kalsotra A, Cooper TA, Johnson JM (2008). Expression of 24,426 human alternative splicing events and predicted cis regulation in 48 tissues and cell lines. Nat Genet.

[6] Matera AG, Wang Z (2014). A day in the life of the spliceosome. Nat Rev Mol Cell Biol.

[7] Szeszel-Fedorowicz W, Talukdar I, Griffith BN, Walsh CM, Salati LM (2006). An exonic splicing silencer is involved in the regulated splicing of glucose 6-phosphate dehydrogenase mRNA. J Biol Chem.

[8] Bland CS, Wang ET, Vu A, David MP, Castle JC, Johnson JM, Burge CB, Cooper TA (2010). Global regulation of alternative splicing during myogenic differentiation. Nucleic Acids Res.

[9] Huelga SC, Vu AQ, Arnold JD, Liang TY, Liu PP, Yan BY, Donohue JP, Shiue L, Hoon S, Brenner S (2012). Integrative genome-wide analysis reveals cooperative regulation of alternative splicing by hnRNP proteins. Cell Rep.

[10] Sebbag-Sznajder N, Raitskin O, Angenitzki M, Sato TA, Sperling J, Sperling R (2012). Regulation of alternative splicing within the supraspliceosome. J Struct Biol.

[11] Sanidas I, Polytarchou C, Hatziapostolou M, Ezell SA, Kottakis F, Hu L, Guo A, Xie J, Comb MJ, Iliopoulos D, Tsichlis PN (2014). Phosphoproteomics screen reveals akt isoform-specific signals linking RNA processing to lung cancer. Mol Cell.

[12] Zhou Z, Qiu J, Liu W, Zhou Y, Plocinik RM, Li H, Hu Q, Ghosh G, Adams JA, Rosenfeld MG, Fu XD (2012). The Akt-SRPK-SR axis constitutes a major pathway in transducing EGF signaling to regulate alternative splicing in the nucleus. Mol Cell.

[13] Zhu C, Yin Z, Tan B, Guo W (2017). Insulin regulates titin pre-mRNA splicing through the PI3K-Akt-mTOR kinase axis in a RBM20-dependent manner. Biochim Biophys Acta.

[14] Blaustein M, Pelisch F, Tanos T, Munoz MJ, Wengier D, Quadrana L, Sanford JR, Muschietti JP, Kornblihtt AR, Caceres JF (2005). Concerted regulation of nuclear and cytoplasmic activities of SR proteins by AKT. Nat Struct Mol Biol.

[15] Shi Y, Reddy B, Manley JL (2006). PP1/PP2A phosphatases are required for the second step of pre-mRNA splicing and target specific snRNP proteins. Mol Cell.

[16] Chalfant CE, Ogretmen B, Galadari S, Kroesen BJ, Pettus BJ, Hannun YA (2001). FAS activation induces dephosphorylation of SR proteins; dependence on the de novo generation of ceramide and activation of protein phosphatase 1. J Biol Chem.

[17] Michlewski G, Sanford JR, Caceres JF (2008). The splicing factor SF2/ASF regulates translation initiation by enhancing phosphorylation of 4E-BP1. Mol Cell.

[18] Johnston JR, Chase PB, Pinto JR (2018). Troponin through the looking-glass: emerging roles beyond regulation of striated muscle contraction. Oncotarget.

[19] Black AJ, Ravi S, Jefferson LS, Kimball SR, Schilder RJ (2017). Dietary fat quantity and type induce transcriptome-wide effects on alternative splicing of pre-mRNA in rat skeletal muscle. J Nutr.

[20] Miklosz A, Lukaszuk B, Baranowski M, Gorski J, Chabowski A (2013). Effects of inhibition of serine palmitoyltransferase (SPT) and sphingosine kinase 1 (SphK1) on palmitate induced insulin resistance in L6 myotubes. PLoS One.

[21] Blachnio-Zabielska AU, Chacinska M, Vendelbo MH, Zabielski P (2016). The crucial role of C18-Cer in fat-induced skeletal muscle insulin resistance. Cell Physiol Biochem.

[22] Nikolova-Karakashian MN, Reid MB (2011). Sphingolipid metabolism, oxidant signaling, and contractile function of skeletal muscle. Antioxid Redox Signal.

[23] Ghosh N, Patel N, Jiang K, Watson JE, Cheng J, Chalfant CE, Cooper DR (2007). Ceramide-activated protein phosphatase involvement in insulin resistance via Akt, serine/arginine-rich protein 40, and ribonucleic acid splicing in L6 skeletal muscle cells. Endocrinology.

[24] Chalfant CE, Rathman K, Pinkerman RL, Wood RE, Obeid LM, Ogretmen B, Hannun YA (2002). De novo ceramide regulates the alternative splicing of caspase 9 and Bcl-x in A549 lung adenocarcinoma cells. Dependence on protein phosphatase-1. J Biol Chem.

[25] Brandimarti P, Costa-Junior JM, Ferreira SM, Protzek AO, Santos GJ, Carneiro EM, Boschero AC, Rezende LF (2013). Cafeteria diet inhibits insulin clearance by reduced insulin-degrading enzyme expression and mRNA splicing. J Endocrinol.

[26] Kim Y, Tamura T, Iwashita S, Tokuyana K, Suzuki M (1994). Effect of high-fat diet on gene expression of GLUT4 and insulin receptor in soleus muslce. Biochem Biophys Res Commun.

[27] Marden JH, Fescemyer HW, Saastamoinen M, MacFarland SP, Vera JC, Frilander MJ, Hanski I (2008). Weight and nutrition affect pre-mRNA splicing of a muscle gene associated with performance, energetics and life history. J Exp Biol.

[28] Tao H, Szeszel-Fedorowicz W, Amir-Ahmady B, Gibson MA, Stabile LP, Salati LM (2002). Inhibition of the splicing of glucose-6-phosphate dehydrogenase precursor mRNA by polyunsaturated fatty acids. J Biol Chem.

[29] Walsh CM, Suchanek AL, Cyphert TJ, Kohan AB, Szeszel-Fedorowicz W, Salati LM (2013). Serine arginine splicing factor 3 is involved in enhanced splicing of glucose-6-phosphate dehydrogenase RNA in response to nutrients and hormones in liver. J Biol Chem.

[30] Griffith BN, Walsh CM, Szeszel-Fedorowicz W, Timperman AT, Salati LM (2006). Identification of hnRNPs K, L and A2/B1 as candidate proteins involved in the nutritional regulation of mRNA splicing. Biochim Biophys Acta.

[31] Kaminska D, Hämäläinen M, Cederberg H, Käkelä P, Venesmaa S, Miettinen P, Ilves I, Herzig KH, Kolehmainen M, Karhunen L (2014). Adipose tissue INSR splicing in humans associates with fasting insulin level and is regulated by weight loss. Diabetologia.

[32] Kaminska D, Kuulasmaa T, Venesmaa S, Käkelä P, Vaittinen M, Pulkkinen L, Pääkkönen M, Gylling H, Laakso M, Pihlajamäki J (2012). Adipose tissue TCF7L2 splicing is regulated by weight loss and associates with glucose and fatty acid metabolism. Diabetes.

[33] Kaminska D, Pihlajamaki J (2013). Regulation of alternative splicing in obesity and weight loss. Adipocyte.

[34] Schilder RJ, Kimball SR, Marden JH, Jefferson LS (2011). Body weight-dependent troponin T alternative splicing is evolutionarily conserved from insects to mammals and is partially impaired in skeletal muscle of obese rats. J Exp Biol.

[35] Webster NJG, Huang Z, Chew SL (1999). Hormonal regulation of alternative splicing. Post-transcriptional processing and the endocrine system.

[36] Schilder RJ, Kimball SR, Jefferson LS (2012). Cell-autonomous regulation of fast troponin T pre-mRNA alternative splicing in response to mechanical stretch. Am J Physiol Cell Physiol..

[37] Black AJ, Gordon BS, Dennis MD, Jefferson LS, Kimball SR (2016). Regulation of protein and mRNA expression of the mTORC1 repressor REDD1 in response to leucine and serum. Biochem Biophys Rep.

[38] Livak KJ, Schmittgen TD (2001). Analysis of relative gene expression data using real-time quantitative PCR and the 2(−Delta Delta C(T)) method. Methods.

[39] Cabane C, Englaro W, Yeow K, Ragno M, Derijard B (2003). Regulation of C2C12 myogenic terminal differentiation by MKK3/p38a pathway. Am J Physiol Cell Physiol.

[40] Perry DK, Carton J, Shah AK, Meredith F, Uhlinger DJ, Hannun YA (2000). Serine Palmitoyltransferase regulates de novo ceramide generation during etoposide-induced apoptosis. J Biol Chem.

[41] Mahfouz R, Khoury R, Blachnio-Zabielska A, Turban S, Loiseau N, Lipina C, Stretton C, Bourron O, Ferre P, Foufelle F (2014). Characterising the inhibitory actions of ceramide upon insulin signaling in different skeletal muscle cell models: a mechanistic insight. PLoS One.

[42] Xu W, Yuan X, Jung YJ, Yang Y, Basso A, Rosen N, Chung EJ, Trepel J, Neckers L (2003). The heat shock protein 90 inhibitor Geldanamycin and the ErbB inhibitor ZD1839 promote rapid PP1 phosphatase-dependent inactivation of AKT in ErbB2 overexpressing breast Cancer cells. Cancer Res.

[43] Holen I, Gordon PB, Seglen PO (1993). Inhibition of hepatocytic autophagy by okadaic acid and other protein phosphatase inhibitors. Eur J Biochem.

[44] Hernandez F, Langa E, Cuadros R, Avila J, Villanueva N (2010). Regulation of GSK3 isoforms by phosphatases PP1 and PP2A. Mol Cell Biochem.

[45] Liu Q, Hofmann PA (2004). Protein phosphatase 2A-mediated cross-talk between p38 MAPK and ERK in apoptosis of cardiac myocytes. Am J Physiol Heart Circ Physiol.

[46] Watt MJ, Barnett AC, Bruce CR, Schenk S, Horowitz JF, Hoy AJ (2012). Regulation of plasma ceramide levels with fatty acid oversupply: evidence that the liver detects and secretes de novo synthesised ceramide. Diabetologia.

[47] Thombare K, Ntika S, Wang X, Krizhanovskii C (2017). Long chain saturated and unsaturated fatty acids exert opposing effects on viability and function of GLP-1-producing cells: mechanisms of lipotoxicity. PLoS One.

[48] Holland WL, Bikman BT, Wang LP, Yuguang G, Sargent KM, Bulchand S, Knotts TA, Shui G, Clegg DJ, Wenk MR (2011). Lipid-induced insulin resistance mediated by the proinflammatory receptor TLR4 requires saturated fatty acid-induced ceramide biosynthesis in mice. J Clin Invest.

[49] Holland WL, Brozinick JT, Wang LP, Hawkins ED, Sargent KM, Liu Y, Narra K, Hoehn KL, Knotts TA, Siesky A (2007). Inhibition of ceramide synthesis ameliorates glucocorticoid-, saturated-fat-, and obesity-induced insulin resistance. Cell Metab.

[50] Zhang Y, Rao E, Zeng J, Hao J, Sun Y, Liu S, Sauter ER, Bernlohr DA, Cleary MP, Suttles J, Li B (2017). Adipose fatty acid binding protein promotes saturated fatty acid-induced macrophage cell death through enhancing ceramide production. J Immunol.

[51] Dobrowsky RT, Kamibayashi C, Mumby MC, Hannun YA (1993). Ceramide activates heterotrimeric protein phosphatase 2A. J Biol Chem.

[52] Chalfant CE, Kishihawa K, Mumby MC, Kamibayashi C, Bielawska A, Hannun YA (1999). Long chain ceramides activate protein phosphatase-1 and protein phosphatase-2A. J Biol Chem.

[53] Chalfant CE, Szulc Z, Roddy P, Bielawska A, Hannun YA (2004). The structural requirements for ceramide activation of serine-threonine protein phosphatases. J Lipid Res.

[54] Glatz JF, Luiken JJ (2015). Fatty acids in cell signaling: historical perspective and future outlook. Prostaglandins Leukot Essent Fatty Acids.

[55] Will CL, Luhrmann R (2005). Splicing of a rare class of introns by the U12-dependent spliceosome. Biol Chem.

[56] Guan Y, Liang G, Martin GB, Guan LL (2017). Functional changes in mRNA expression and alternative pre-mRNA splicing associated with the effects of nutrition on apoptosis and spermatogenesis in the adult testis. BMC Genomics.

[57] Ravi S, Schilder RJ, Berg AS, Kimball SR (2016). Effects of age and hindlimb immobilization and remobilization on fast troponin T precursor mRNA alternative splicing in rat gastrocnemius muscle. Appl Physiol Nutr Metab.

