# Supplementary material for: Palmitate- and C6 ceramide-induced Tnnt3 pre-mRNA alternative splicing occurs in a PP2A dependent manner
Source: Nutr Metab (Lond). 2018 Dec 17;15:87. doi: 10.1186/s12986-018-0326-3 (PMC6296074; doi:10.1186/s12986-018-0326-3)
Supplement: Supplementary file 1 — Fold change in the relative abundance of Tnnt3 splice forms in L6 myotubes treated with C6 ceramide. (DOCX 15 kb) [file 12986_2018_326_MOESM1_ESM.docx]

Additional File 1. Fold change in the relative abundance of *Tnnt3* splice forms

| *Tnnt3*  splice form  size (bp) | Vehicle | C6 Ceramide |
| --- | --- | --- |
| 710 | 1.00 | 1.780 ± 0.424 |
| 725 | 1.00 | 1.237 ± 0.157 |
| 728 | 1.00 | 0.966 ± 0.076 |
| 737 | 1.00 | 2.792 ± 0.516 * |
| 739 | 1.00 | 1.445 ± 0.192 |
| 742 | 1.00 | 0.737 ± 0.081 * |
| 751 | 1.00 | 3.001 ± 0.338 * |
| 754 | 1.00 | 1.100 ± 0.418 |
| 757 | 1.00 | 0.972 ± 0.110 |
| 763 | 1.00 | 1.406 ± 0.303 |
| 769 | 1.00 | 1.000 ± 0.224 |
| 775 | 1.00 | 3.133 ± 0.969 |
| 778 | 1.00 | 1.451 ± 0.246 |
| 781 | 1.00 | 0.760 ± 0.055 * |
| 790 | 1.00 | 1.745 ± 0.232 * |
| 793 | 1.00 | 0.907 ± 0.136 |
| 795 | 1.00 | 0.872 ± 0.060 |
| 807 | 1.00 | 0.796 ± 0.030 * |

L6 myotubes were treated for 24 hours with 20 µM C6 ceramide or an equal volume of methanol (Vehicle). The fold change in the relative abundance of *Tnnt3* splice forms was assessed by capillary electrophoresis. Data are presented as means ± SEM from three independent experiments using three replicates per treatment. Statistically different means as assessed by Student’s t-test are denoted with an asterisk (*) (p ≤ 0.05).
