# Supplementary material for: Palmitate- and C6 ceramide-induced Tnnt3 pre-mRNA alternative splicing occurs in a PP2A dependent manner
Source: Nutr Metab (Lond). 2018 Dec 17;15:87. doi: 10.1186/s12986-018-0326-3 (PMC6296074; doi:10.1186/s12986-018-0326-3)
Supplement: Supplementary file 2 — Fold change in the relative abundance of Tnnt3 splice forms in L6 myotubes treated with myriocin. (DOCX 16 kb) [file 12986_2018_326_MOESM2_ESM.docx]

Additional File 2. Fold change in the relative abundance of *Tnnt3* splice forms

|  | BSA | |  | PA | |
| --- | --- | --- | --- | --- | --- |
| *Tnnt3*  splice form  size (bp) | Vehicle | Myriocin |  | Vehicle | Myriocin |
| 710 | 1.00 ^a^ | 0.927 ± 0.146 ^a^ |  | 0.904 ± 0.080 ^a^ | 0.755 ± 0.145 ^a^ |
| 725 | 1.00 ^a^ | 0.792 ± 0.022 ^b^ |  | 0.876 ± 0.036 ^c^ | 0.889 ± 0.015 ^c^ |
| 728 | 1.00 ^a^ | 1.096 ± 0.059 ^a^ |  | 0.967 ± 0.142 ^a^ | 1.171 ± 0.167 ^a^ |
| 737 | 1.00 ^a^ | 1.178 ± 0.137 ^a^ |  | 1.529 ± 0.128 ^b^ | 0.958 ± 0.064 ^a^ |
| 739 | 1.00 ^a^ | 0.826 ± 0.071 ^a^ |  | 0.838 ± 0.153 ^a^ | 0.863 ± 0.101 ^a^ |
| 742 | 1.00 ^a^ | 1.258 ± 0.171 ^a^ |  | 1.089 ± 0.037 ^a^ | 1.085 ± 0.167 ^a^ |
| 751 | 1.00 ^a^ | 1.231 ± 0.248 ^a^ |  | 0.969 ± 0.047 ^a^ | 1.030 ± 0.207 ^a^ |
| 754 | 1.00 ^a^ | 1.259 ± 0.183 ^a^ |  | 1.193 ± 0.216 ^a^ | 1.058 ± 0.025 ^a^ |
| 757 | 1.00 ^a^ | 0.994 ± 0.074 ^a^ |  | 0.952 ± 0.062 ^a^ | 1.126 ± 0.080 ^a^ |
| 763 | 1.00 ^a^ | 0.936 ± 0.017 ^a^ |  | 0.984 ± 0.029 ^a^ | 1.111 ± 0.034 ^b^ |
| 769 | 1.00 ^a^ | 1.109 ± 0.081 ^a^ |  | 1.111 ± 0.130 ^a^ | 1.074 ± 0.066 ^a^ |
| 775 | 1.00 ^a^ | 0.769 ± 0.148 ^a^ |  | 0.991 ± 0.165 ^a^ | 0.725 ± 0.136 ^a^ |
| 778 | 1.00 ^a^ | 0.985 ± 0.114 ^a^ |  | 1.084 ± 0.080 ^a^ | 0.950 ± 0.096 ^a^ |
| 781 | 1.00 ^a^ | 0.995 ± 0.037 ^a^ |  | 0.983 ± 0.024 ^a^ | 0.975 ± 0.020 ^a^ |
| 790 | 1.00 ^a^ | 0.986 ± 0.067 ^a^ |  | 1.039 ± 0.030 ^a^ | 1.069 ± 0.124 ^a^ |
| 793 | 1.00 ^a^ | 0.953 ± 0.065 ^a^ |  | 0.980 ± 0.102 ^a^ | 1.139 ± 0.109 ^a^ |
| 795 | 1.00 ^a^ | 1.002 ± 0.037 ^a^ |  | 1.017 ± 0.007 ^a^ | 0.973 ± 0.018 ^a^ |
| 807 | 1.00 ^a^ | 0.958 ± 0.036 ^a^ |  | 0.940 ± 0.016 ^a^ | 0.972 ± 0.013 ^a^ |

L6 myotubes were pretreated for two hours with 50 nM myriocin or an equal volume of methanol (Vehicle) prior to a 24-hour treatment with 150 µM palmitate conjugated to BSA (PA) or an equal volume of BSA alone. The fold change in the relative abundance of *Tnnt3* splice forms was assessed by capillary electrophoresis. Data are presented as means ± SEM from three independent experiments using three replicates per treatment. Statistical significance was assessed by One-way ANOVA with Fishers LSD post-hoc test for multiple comparisons. Statistically different means are denoted with different letters (p ≤ 0.05).
