# Supplementary material for: Palmitate- and C6 ceramide-induced Tnnt3 pre-mRNA alternative splicing occurs in a PP2A dependent manner
Source: Nutr Metab (Lond). 2018 Dec 17;15:87. doi: 10.1186/s12986-018-0326-3 (PMC6296074; doi:10.1186/s12986-018-0326-3)
Supplement: Supplementary file 3 — Fold change in the relative abundance of Tnnt3 splice forms in L6 myotubes treated with okadaic acid and palmitate. (DOCX 16 kb) [file 12986_2018_326_MOESM3_ESM.docx]

Additional File 3. Fold change in the relative abundance of *Tnnt3* splice forms

|  | BSA | |  | PA | |  |
| --- | --- | --- | --- | --- | --- | --- |
| *Tnnt3*  splice form  size (bp) | Vehicle | Okadaic Acid |  | Vehicle | Okadaic Acid |  |
| 710 | 1.00 ^a^ | 1.057 ± 0.226 ^a^ |  | 0.921 ± 0.041 ^a^ | 1.022 ± 0.135 ^a^ |  |
| 725 | 1.00 ^a^ | 1.132 ± 0.028 ^a^ |  | 0.962 ± 0.036 ^a^ | 1.111 ± 0.207 ^a^ |  |
| 728 | 1.00 ^a^ | 1.845 ± 0.108 ^b^ |  | 1.267 ± 0.251 ^a^ | 1.330 ± 0.111 ^a^ |  |
| 737 | 1.00 ^a^ | 0.564 ± 0.070 ^b^ |  | 1.147 ± 0.048 ^a*^ | 0.597 ± 0.060 ^b^ |  |
| 739 | 1.00 ^a^ | 0.996 ± 0.062 ^a^ |  | 0.826 ± 0.134 ^a^ | 1.026 ± 0.061 ^a^ |  |
| 742 | 1.00 ^a^ | 1.120 ± 0.224 ^a^ |  | 0.942 ± 0.120 ^a^ | 1.298 ± 0.147 ^a^ |  |
| 751 | 1.00 ^a^ | 0.773 ± 0.112 ^b^ |  | 1.321 ± 0.119 ^c^ | 0.720 ± 0.050 ^b^ |  |
| 754 | 1.00 ^a^ | 0.655 ± 0.026 ^b^ |  | 1.511 ± 0.083 ^c^ | 0.564 ± 0.115 ^b^ |  |
| 757 | 1.00 ^a^ | 1.203 ± 0.074 ^a^ |  | 1.037 ± 0.126 ^a^ | 1.337 ± 0.128 ^b^ |  |
| 763 | 1.00 ^a, b^ | 1.338 ± 0.092 ^c^ |  | 0.861 ± 0.036 ^a^ | 1.199 ± 0.085 ^b, c^ |  |
| 769 | 1.00 ^a^ | 0.609 ± 0.052 ^b^ |  | 1.145 ± 0.112 ^a^ | 0.701 ± 0.036 ^b^ |  |
| 775 | 1.00 ^a^ | 1.039 ± 0.137 ^a^ |  | 1.703 ± 0.209 ^b^ | 1.143 ± 0.141 ^a^ |  |
| 778 | 1.00 ^a^ | 1.567 ± 0.047 ^b^ |  | 1.225 ± 0.109 ^c^ | 1.686 ± 0.081 ^b^ |  |
| 781 | 1.00 ^a^ | 1.071 ± 0.010 ^b^ |  | 0.983 ± 0.017 ^a^ | 0.994 ± 0.016 ^a^ |  |
| 790 | 1.00 ^a^ | 0.724 ± 0.064 ^c^ |  | 0.909 ± 0.025 ^a, b^ | 0.808 ± 0.048 ^b, c^ |  |
| 793 | 1.00 ^a^ | 0.523 ± 0.049 ^b^ |  | 0.865 ± 0.037 ^a^ | 0.538 ± 0.094 ^b^ |  |
| 795 | 1.00 ^a^ | 1.526 ± 0.073 ^b^ |  | 1.019 ± 0.066 ^a^ | 1.498 ± 0.075 ^b^ |  |
| 807 | 1.00 ^a^ | 0.799 ± 0.033 ^b^ |  | 0.954 ± 0.027 ^a^ | 0.823 ± 0.040 ^b^ |  |

L6 myotubes were pretreated for two hours with 15 nM okadaic acid or an equal volume of DMSO (Vehicle) prior to a 24-hour treatment of 150 µM palmitate conjugated to BSA (PA) or an equal volume of BSA alone. The fold change in the relative abundance of *Tnnt3* splice forms was assessed by capillary electrophoresis. Data are presented as means ± SEM from three independent experiments using three replicates per treatment. Statistical significance was assessed by Two-way ANOVA with Fishers LSD post-hoc test for multiple comparisons. Statistically different means are denoted with different letters (p ≤ 0.05). * p = 0.08 vs. BSA/Vehicle
