# Supplementary material for: Palmitate- and C6 ceramide-induced Tnnt3 pre-mRNA alternative splicing occurs in a PP2A dependent manner
Source: Nutr Metab (Lond). 2018 Dec 17;15:87. doi: 10.1186/s12986-018-0326-3 (PMC6296074; doi:10.1186/s12986-018-0326-3)
Supplement: Supplementary file 4 — Fold change in the relative abundance of Tnnt3 splice forms in L6 myotubes treated with okadaic acid and C6 ceramide. (DOCX 16 kb) [file 12986_2018_326_MOESM4_ESM.docx]

Additional File 4. Fold change in the relative abundance of *Tnnt3* splice forms

|  | MeOH | |  | C6 Ceramide | |
| --- | --- | --- | --- | --- | --- |
| *Tnnt3*  splice form  size (bp) | Vehicle | Okadaic Acid |  | Vehicle | Okadaic Acid |
| 710 | 1.00 ^a^ | 1.326 ± 0.304 ^a^ |  | 1.190 ± 0.235 ^a^ | 1.190 ± 0.346 ^a^ |
| 725 | 1.00 ^a^ | 1.054 ± 0.167 ^a^ |  | 1.161 ± 0.112 ^a^ | 1.304 ± 0.224 ^a^ |
| 728 | 1.00 ^a, b^ | 1.220 ± 0.183 ^a^ |  | 0.581 ± 0.066 ^b^ | 0.907 ± 0.202 ^a, b^ |
| 737 | 1.00 ^a^ | 0.389 ± 0.049 ^a^ |  | 2.154 ± 0.487 ^b^ | 0.858 ± 0.227 ^a^ |
| 739 | 1.00 ^a, b^ | 0.723 ± 0.145 ^a^ |  | 1.167 ± 0.146 ^b^ | 0.850 ± 0.122 ^a, b^ |
| 742 | 1.00 ^a^ | 1.333 ± 0.300 ^a^ |  | 0.840 ± 0.046 ^a^ | 1.229 ± 0.299 ^a^ |
| 751 | 1.00 ^a^ | 0.401 ± 0.021 ^a^ |  | 2.186 ± 0.535 ^b^ | 0.832 ± 0.159 ^a^ |
| 754 | 1.00 ^a^ | 0.740 ± 0.071 ^a^ |  | 1.644 ± 0.435 ^a^ | 1.598 ± 0.428 ^a^ |
| 757 | 1.00 ^a^ | 1.009 ± 0.154 ^a^ |  | 0.980 ± 0.131 ^a^ | 1.101 ± 0.207 ^a^ |
| 763 | 1.00 ^a^ | 1.106 ± 0.227 ^a^ |  | 0.973 ± 0.144 ^a^ | 0.964 ± 0.065 ^a^ |
| 769 | 1.00 ^a^ | 0.482 ± 0.044 ^b^ |  | 1.358 ± 0.119 ^c^ | 1.003 ± 0.154 ^a^ |
| 775 | 1.00 ^a^ | 0.951 ± 0.169 ^a^ |  | 1.159 ± 0.320 ^a^ | 0.664 ± 0.395 ^a^ |
| 778 | 1.00 ^a^ | 1.559 ± 0.023 ^b, c^ |  | 1.294 ± 0.055 ^a, c^ | 1.761 ± 0.172 ^b^ |
| 781 | 1.00 ^a^ | 1.245 ± 0.064 ^b^ |  | 0.897 ± 0.035 ^a^ | 1.016 ± 0.043 ^a^ |
| 790 | 1.00 ^a, b^ | 0.746 ± 0.116 ^a^ |  | 1.509 ± 0.325 ^b^ | 0.901 ± 0.297 ^a, b^ |
| 793 | 1.00 ^a^ | 0.675 ± 0.128 ^a, b^ |  | 0.512 ± 0.152 ^b^ | 0.572 ± 0.085 ^b^ |
| 795 | 1.00 ^a^ | 1.291 ± 0.057 ^b^ |  | 0.909 ± 0.063 ^a^ | 1.100 ± 0.089 ^a, c^ |
| 807 | 1.00 ^a^ | 0.743 ± 0.068 ^a, b^ |  | 0.748 ± 0.101 ^a, b^ | 0.682 ± 0.119 ^b^ |

L6 myotubes were pretreated for two hours with 15 nM okadaic acid or an equal volume of DMSO (Vehicle) prior to a 24-hour treatment of 20 µM C6 or an equal volume of methanol (MeOH). The fold change in the relative abundance of *Tnnt3* splice forms was assessed by capillary electrophoresis. Data are presented as means ± SEM from three independent experiments using three replicates per treatment. Statistical significance was assessed by Two-way ANOVA with Fishers LSD post-hoc test for multiple comparisons. Statistically different means are denoted with different letters (p ≤ 0.05).
